# Comparative Study of Green Sub- and Supercritical Processes to Obtain Carnosic Acid and Carnosol-Enriched Rosemary Extracts with in Vitro Anti-Proliferative Activity on Colon Cancer Cells

**DOI:** 10.3390/ijms17122046

**Published:** 2016-12-07

**Authors:** Andrea del Pilar Sánchez-Camargo, Virginia García-Cañas, Miguel Herrero, Alejandro Cifuentes, Elena Ibáñez

**Affiliations:** Laboratory of Foodomics, Institute of Food Science Research, Instituto de Investigación en Ciencias de la Alimentación, Consejo Superior de Investigaciones Científicas, Nicolas Cabrera 9, 28049 Madrid, Spain; andreap.sanchez@csic.es (A.d.P.S.-C.); virginia.garcia@csic.es (V.G.-C.); m.herrero@csic.es (M.H.); a.cifuentes@csic.es (A.C.)

**Keywords:** rosemary, supercritical fluid extraction (SFE), process intensification, subcritical fluids, supercritical fluids, anti-proliferative, colon cancer cell, HT-29, HCT116

## Abstract

In the present work, four green processes have been compared to evaluate their potential to obtain rosemary extracts with in vitro anti-proliferative activity against two colon cancer cell lines (HT-29 and HCT116). The processes, carried out under optimal conditions, were: (1) pressurized liquid extraction (PLE, using an hydroalcoholic mixture as solvent) at lab-scale; (2) Single-step supercritical fluid extraction (SFE) at pilot scale; (3) Intensified two-step sequential SFE at pilot scale; (4) Integrated PLE plus supercritical antisolvent fractionation (SAF) at pilot scale. Although higher extraction yields were achieved by using PLE (38.46% dry weight), this extract provided the lowest anti-proliferative activity with no observed cytotoxic effects at the assayed concentrations. On the other hand, extracts obtained using the PLE + SAF process provided the most active rosemary extracts against both colon cancer cell lines, with LC_50_ ranging from 11.2 to 12.4 µg/mL and from 21.8 to 31.9 µg/mL for HCT116 and HT-29, respectively. In general, active rosemary extracts were characterized by containing carnosic acid (CA) and carnosol (CS) at concentrations above 263.7 and 33.9 mg/g extract, respectively. Some distinct compounds have been identified in the SAF extracts (rosmaridiphenol and safficinolide), suggesting their possible role as additional contributors to the observed strong anti-proliferative activity of CA and CS in SAF extracts.

## 1. Introduction

Nowadays, supercritical fluid extraction (SFE) employing CO_2_ is an established industrial process for the production of high added-value products. By 2014, there were more than 150 SFE industrial plants with a total extraction volume of more than 500 L around the world. Many of these production plants are generally devoted to the SFE of natural products, leading to the recovery of high-value products which provide interesting options for their use in the nutraceutical and functional food industry [1]. In this sense, the search for bioactive compounds or “target molecules” from natural sources (marine-derived and agro-industrial products or their by-products) has become the most important application of the scientific research of SFE [2,3,4,5,6,7,8]. According to the review work recently published by Da Silva et al. (2016), the bioactivities from natural compounds obtained by SFE from 2010 to 2015 were mainly antioxidant (41%), antitumor (18%) and antibacterial (10%), followed by antiviral, antimicrobial, anti-inflammatory and anticholinesterase activity (in a total of 5%) [2]. In this regard, the recovery of bioactive compounds from herbs and spices (especially those belonging to the *Lamiaceae* family) employing supercritical and subcritical fluids has been intensified in the last 10 years [5,9,10], mainly due to several biological properties such as: antioxidant [11,12,13], antimicrobial [14,15,16,17], anti-proliferative [18,19], antitumor [20,21], anti-inflammatory [22,23,24] and anti-obesity [25,26], among others. Rosemary-leaf extracts (*Rosmarinus officinalis* L.) have received special attention since European Food Safety Authority and U.S. Food and Drug Administration approved their use as food additive, demonstrating to be safe for human health at specific compositions [27,28]. Several rosemary compounds, principally carnosol (CS) [29,30,31,32], carnosic acid (CA) [33,34,35], and ursolic acid [36,37,38], have demonstrated different anticancer activities, such as anti-proliferative, antiinvasive, and antitumorigenic effects, in a dose-dependent manner. Interestingly, although several of the observed anti-proliferative effects of rosemary extracts have been commonly attributed to some of these components, in many cases the effect of the complete extract is higher than that exerted by the individual compounds [20,21,30,33,39,40]. In some cases, this general observation has been attributed to the synergistic effect of the combination of the main bioactive compounds with other unknown and minor compounds present in the extract [20,21]. These functional aspects of rosemary extracts make necessary to standardize the extraction methodology to achieve an extract composition that exhibits the pursued anti-proliferative activity. Some authors have demonstrated the superior anti-proliferative activity of the SFE rosemary extracts compared to aqueous and methanolic extracts in leukemia, lung, liver, prostate, breast and colon cancer cells [18,33]. In our laboratory, the investigation of green processes extraction methodologies has mainly been focused on the concentration enhancement of bioactive compounds in rosemary extracts by means of multistage fractionation or combination of sub- and supercritical fluid methodologies with upstream process optimization [41,42,43]. These strategies are in good agreement with the recent trends directed to the development of new intensified and integrated processes, which seems to be more suitable for complex vegetable matrixes such as rosemary [1,42,44,45]. In process intensification, the same multipurpose equipment is used for different unit operations, while in the integrated process, the best process for obtaining each product is sought, where different equipment is commonly employed [44]. The process intensification concept using supercritical fluids has been recently applied in several areas as an option for the future production of substitutes for petrochemical derivatives from biomass, mainly as source of energy and biofuels [46,47]. We recently reported the use of a pilot scale SFE equipment to study a two-sequential step SFE as intensification process. Following this strategy, it was possible to obtain two fractions, one rich in volatile oil (containing 1,8-cineole and camphor) and the other rich in CA and CS, being this latter fraction tested for its inhibitory activity against colon cancer cell proliferation [42]. On the other hand, process integration includes prior unit operation (fermentation, extraction, enzyme pre-treatment, physical fractionation or size reduction) followed by sub-or supercritical extraction or fractionation processes (supercritical chromatography, enzymatic conversion, precipitation and coating of solutes, among others) [45]. Regarding this approach, recent reports suggest the combined use of extraction processes, such as supercritical CO_2_ followed by pressurized liquid extraction (PLE, employing ethanol and water) for improving the recovery of compounds with different polarities and bio-functionalities [44,48,49,50]. Another exceptionally versatile process that has been used in the integrated SF-processes is the Supercritical Antisolvent Fractionation (SAF), which benefits from the antisolvent properties of supercritical carbon dioxide (SC-CO_2_) and allows for the precipitation of insoluble compounds in the SC-CO_2_ organic solution mixture [45,51]. Following these ideas, an integration of two lab-scale processes, PLE and SAF was carried out to obtain two fractions enriched in different families of compounds: a raffinate fraction (enriched in phenolic acids, mainly rosmarinic acid) and an extract fraction (enriched in phenolic diterpenes, mainly CA and CS) [41]. The integrated process was optimized and the extract fraction showed improved in vitro anti-proliferative activity against human colon adenocarcinoma cells. Once the processes have been optimized at lab-scale, it is mandatory to test their efficacy at large scale; this step is crucial for the future implementation of standardized processes leading to a product with well-known composition and bioactivity.

Therefore, in the present work, a comparative study of the four types of green processes previously developed at lab-scale was carried out to evaluate their potential to obtain rosemary extracts with in vitro anti-proliferative activity against two colon cancer cell lines (HT-29 and HCT116). The comparison was performed on several rosemary extracts obtained by PLE using an hydroalcoholic mixture as solvent with standard lab-scale equipment (a); and three different processes at pilot scale, namely, single-step SFE (b), two-step sequential SFE (process intensification, c), and PLE + SAF (process integration, d). To achieve that, PLE + SAF process (d) was assessed at higher scale in the present study, and the resulting extracts were compared in terms of yield, chemical composition, and antioxidant and anti-proliferative activities to the extracts obtained by the other three previously optimized processes (a–c). All the rosemary extracts were chemically characterized using different MS-based analytical techniques in an attempt to correlate the presence of specific rosemary constituents with the observed bioactivities.

## 2. Results and Discussion

### 2.1. Yield of Phenolic Compounds and Antioxidant Properties of the Extracts

In the present study, four green processes were selected based on previous works focused on the enrichment of rosemary extracts on CA and CS [41,42], to investigate their potential for the production of rosemary leaves’ extracts and fractions with potent in vitro anti-proliferative activity against colon cancer cells. Thus, PLE process was chosen since it is suitable for providing a rosemary extract at lab scale that can be also used to obtain the starting material required for other processes (see below). In addition to this, we selected three different processes performed at pilot scale. Namely, a single-step SFE process using ethanol as co-solvent (7%, *w*/*w*) [41], a two-step SFE process for further CA enrichment of the extract [41] and an integrated process that involves PLE and supercritical antisolvent fractionation [41]. A total of six rosemary extracts were obtained in duplicate using the conditions summarized in Table 1. As mentioned, the SAF process, previously optimized at lab scale to obtain extracts fractions enriched in the phenolic diterpenes [41], was performed at pilot scale in the present work to obtain three different extracts (SAF1-3) to make them comparable with the other extracts obtained at pilot scale. As can be observed in Table 2, PLE process provided the highest extraction yield (38.46 g/100 g rosemary-leaf dry) due mainly to the polarity solvent and the lower selectivity of this procedure. On the other hand, despite the co-solvent and other distinct SFE extraction conditions, the low extraction yields of SFE1 and SFE2 processes were not significantly different. The extraction yields (or recoveries, in this case) of the SAF processes were higher when the feed to SC-CO_2_ mass flow ratio was the lowest. Thus, around 21% (*w*/*w*) of dry PLE extract was recovered employing SAF1 conditions, in accordance with the values obtained at lab-scale [41]. Total phenol values were statistically different among processes studied and did not correlate linearly (at confidence level of 95%) with the antioxidant activity, expressed as Trolox equivalent antioxidant capacity (TEAC) assay (*r* = 0.64) and EC_50_ (half maximal effective concentration) (*r* = −0.23). The highest phenol amount was achieved in PLE sample (233.9 mg Galic acid equivalent (GAE)/g extract), which is in agreement with the nature of the solvent (ethanol/water mixture) employed to extract this kind of compounds. However, this high total phenolic content (TPC) value for PLE extract does not correspond with the highest TEAC and lowest EC_50_ values among the extracts obtained. For instance, SAF1 exhibited a lower TPC value than PLE sample; however, their TEAC and EC_50_ values were 49% higher and 56% lower, respectively. This evidence could suggest that other types of compounds present in this extract had a positive influence on the antioxidant activity. A similar observation can be done for SFE2 and SAF2 extracts. Interestingly, SAF2 and SAF3 showed very similar TEAC values but the concentrations required to inhibit 2,2-diphenyl-1-picrylhydrazyl (DPPH) radical by half were very different among them. A similar discrepancy was observed in the analysis of SFE2 and PLE extracts. In support of these observations, Yesil-Celiktas et al. (2007) reported similar results, indicating that TEAC assay did not show any correlation with phenol content (*r* = −0.17) and DPPH assay (*r* = 0.16), in rosemary extracts obtained by SFE [52]. These phenomena could be explained by the mechanism employed by 2,2′-azino-bis(3-ethylbenzothiazoline-6-sulphonic acid) (ABTS) radical which is not able to allow for discrimination between the genuine antioxidant compounds and just expresses well by reducing agents that react primarily by a single electron transfer mechanism [53]. Comparing the six extracts, the conditions used to obtain SAF1 samples seemed to be the most adequate green alternative in order to maximize total phenolic content and antioxidant activity. The potency of all the extracts to inhibit colon cancer cell proliferation was next tested on HT-29 and HCT116 cell lines.

### 2.2. Anti-Proliferative Activity of the Extracts

**Figure 1:**
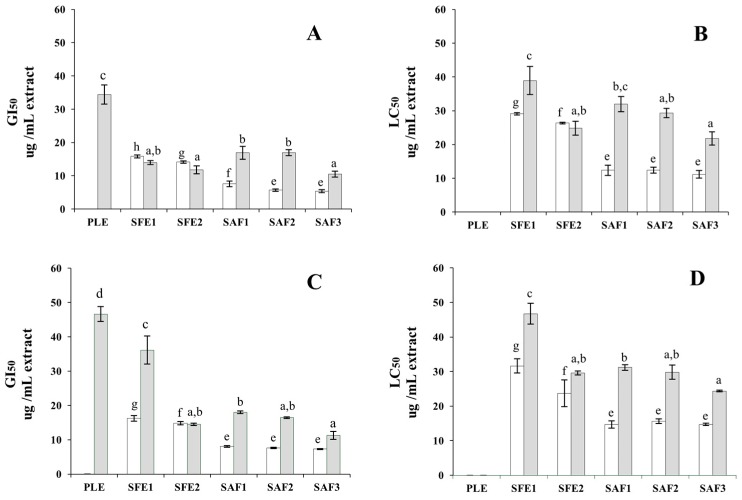
Cytostatic (**A**,**C**) and cytotoxic (**B**,**D**) activities of the rosemary extract on HCT116 (white) and HT-29 cells (grey) at different exposure times. Calculated GI_50_ values at 24 h (**A**) and 72 h (**C**); calculated LC_50_ values at 24 h (**B**) and 72 h (**D**). Error bars represent standard error of the mean (SEM). In each bar graph, mean values that do not share superscripts letters indicate that they differ by *p* < 0.05 as analyzed by one-way analysis of variance (ANOVA). Superscript letters (e–h) and (a–d) have been used to indicate ANOVA results in HCT116 and HT-29 cell lines, respectively.

To determine the anti-proliferative effect of the polyphenol-enriched extracts, HT-29 and HCT116 cells were incubated with increasing concentrations of extracts (from 0 to 50 µg/ mL) for 24 and 72 h and cell proliferation was analyzed by the 3-(4,5-dimethylthiazol-2-yl)-2,5-diphenyltetrazolium bromide (MTT) assay. All the extracts exhibited a concentration-dependent anti-proliferative effect after both exposure times (Figure S1, Supplementary Materials). In order to characterize the anti-proliferative activity of these extracts in more detail and compare their potencies, the growth inhibition (GI_50_, as an indicator for cytostaticity) and the lethal concentration (LC_50_, as an indicator for cytotoxicity), were also determined in both cell lines in time course experiments at 24 and 72 h incubation times. As it is shown in Figure 1, HT-29 and HCT116 cell lines showed different sensitivity to the extracts. Specifically, HCT116 cells were less refractory to the inhibitory and cytotoxic effect of the three SAF extracts than HT-29 cells. Thus, these particular extracts exhibited good cytostatic potential at concentrations below 10 µg/mL in HCT116 cells after 24 h-exposures, whereas GI_50_ values obtained in the assays with HT-29 cells were above that concentration (Figure 1A). The superior potency of SAF extracts towards HCT116 cells is also illustrated in Figure 1B, where it is shown that extract concentrations ranging from 11.2 to 12.4 µg/mL were sufficient to induce 50% HCT116 cell death (LC_50_), whereas higher concentrations (from 21.8 to 31.9 µg/mL) were needed to exert a comparable cytotoxic effect in the HT-29 cell line. To note, the activity of the three SAF extracts was statistically comparable in HCT116 cells, whereas one of them, SAF3 extract, showed to be more effective than the other two extracts against HT-29 cells. Regarding the latter cell line, it showed an extraordinary tolerance to cytostatic concentrations of SFE1 extract after 72 h-exposure (Figure 1C). In general, the inhibitory effects of the extracts did not significantly vary by the exposure time (Figure 1C,D). An exception to this was observed with SFE1 extract whose effect was significantly stronger in HT-29 cells at 24 h than at 72 h. These results suggest that both cytostatic and cytotoxic concentrations of SFE1 extract allow prompt cell recovery with partial restoration of proliferation. On the other hand, PLE extract exhibited the lowest anti-proliferative activity among the studied extracts, providing GI_50_ values above 30 µg/mL for HCT116 cells whereas GI_50_ values for HT-29 cells were outside the testing range. In addition, PLE extract did not exert cytotoxic effects at the assayed concentrations. In vitro studies have often confirmed strong anti-proliferative effects to be associated with phenolic-rich rosemary extracts [18,33,40,54,55,56]. Interestingly, in the present study, examination of the total phenolic content of the six extracts (Table 2) indicated that there is a lack of positive correlation between the total phenolics in the extracts and their anti-proliferative activity in both cell lines. This observation is illustrated by PLE extract, which showed the highest phenolics content but exhibited the lowest anti-proliferative activity in both cell lines. This lack of correlation suggests that the effect of the extracts on cell proliferation and viability may be due to certain extract constituents. In this regard, the anti-proliferative activity (and also the antioxidant activity) of rosemary extracts has frequently been attributed to the presence of major diterpenes, CA and CS [19,57]. Nevertheless, individual CA and CS in pure solutions or in binary mixtures at the same concentrations as those found in rosemary extracts appear to exert lower inhibitory effects than the whole extracts [20,56]. In our present study, to investigate the selectivity of each process for the extraction of specific bioactive compounds (or groups of compounds) and their potential correlation with the anti-proliferative activity, the chemical compositions of the extracts were assessed using different MS-based analytical techniques.

### 2.3. Chemical Characterization of the Extracts by LC-DAD-MS, LC-Q/TOF-MS, and GC-MS

**Figure 2:**
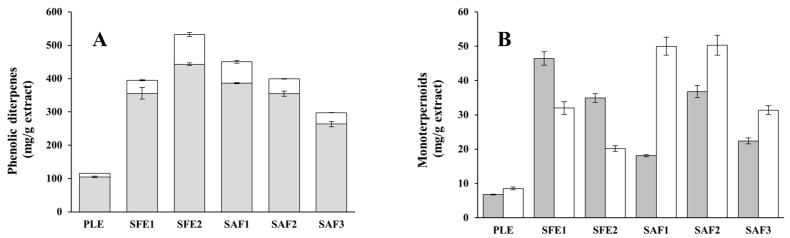
Concentration of main phenolic diterpenes and monoterpenes (mg/g) in the rosemary extracts. (**A**) carnosic acid (CA) (grey) and carnosol (CS) (white); (**B**) 1,8-cineole (grey) and camphor (white). Error bars represent standard deviation (SD).

The quantification of main phenolic (CA and CS, carried out by liquid chromatography coupled to Diode Array Detector and mass spectrometry (LC-DAD-MS)) and volatile compounds (1,8-cineole and camphor, by gas chromatography coupled to mass spectrometry (GC-MS)) in the rosemary extracts is presented in the Supplementary Materials (Table S1). Chromatographic data revealed that high enrichment in CA and CS was achieved in all the extracts, ranging from 263.7 to 443.3 and from 33.9 to 88.9 mg/g extract, respectively (Figure 2A). An exception to this was PLE extract in which CA and CS were only found at 105.0 and 10.7 mg/g extract, respectively. SFE2 extracts was the most enriched in both diterpenes (443.3 and 88.9 mg/g extract, respectively), achieving an improved extract with more than 50% (*w*/*w*) of these phenolic diterpenes in their total composition. Also, similar to the lab scale approach [42], the CA + CS concentration, TPC and antioxidant activity in the pilot SAF scale were found to be in the following order: SAF1 > SAF2 > SAF3. However, in terms of recovery, the lab-scale process showed lower values. On the other hand, a comparison among SAF and the other processes indicated a new order for CA + CS enrichment in the extracts as follows: SFE2 > SAF1 > SAF2 ≥ SFE1 > SAF3 > PLE.

Next, CA and CS concentrations, as well as the sum of both concentrations in the extracts, were compared to their anti-proliferative activity. As expected, the less active extract (PLE) was the one containing the lowest CA and CS concentration. However, excluding the observation for PLE extract, a lack of positive correlation between the extract potency and the CA and CS concentrations was observed in the rest of the rosemary extracts. Interestingly, SAF3 extract was less enriched in CA and CS than those obtained using antisolvent fractionation technology, but was the most active (among SAF extracts) inhibiting cell proliferation and inducing cytotoxic effects in HT-29 cells. Furthermore, SFE1 and SAF2 extracts showed comparable CA and CS concentrations (*p*-value > 0.05; Supplementary Materials, Table S1); however, their activities were strikingly different in both cell lines. These results suggest the potential presence of other unidentified rosemary constituents that may also contribute to the observed anti-proliferative effect of the extracts (especially in SAF3). This lack of positive correlation between the concentration of the two major diterpenes in the extracts and the level of inhibition of cell proliferation prompted us to examine other chemical constituents in the extracts. Thus, UHPLC-qTOF-MS analysis of the extracts was performed to identify potential active compounds present in the extracts. Although all extracts provided similar chromatographic profiles (Supplementary Materials, Figure S2), interesting qualitative and quantitative differences were observed among them. Table 3 summarizes the 29 resolved peaks and 17 compounds that have been tentatively identified. The identified compounds could be classified according to their nature as follows: phenolic acids (syringic acid, rosmarinic acid and tryhydroxycinnamic acid derivate), flavonoids (gallocathechin and genkwanin), phenolic terpenes (rosmanol, epirosmanol/isorosmanol, rosmadial, carnosol, carnosic acid, rosmaridiphenol, methyl carnosate/12-methoxycarnosic acid, betulinic acid, oleanolic acid and ursolic acid), dihydrocoumarins (safficinolide) and diterpene lactones (11,12-dimethylrosmanol). Some works have previously described these families of compounds typically found in rosemary extracts [10,27,43,58,59]. In order to detect if there exists a similarity among extracts with potent anti-proliferative activity, results were examined to identify the compounds that were exclusively present in the most potent extracts compared to those extracts with lower activities. Interestingly, three signals with masses 207.064 (tryhydroxycinnamic acid derivate), 331.156 (NI3) and 315.197 (rosmaridiphenol) Da were only found in the three most potent extracts (SAF1-3), showing maximum peak area values in the chromatographic analysis of SAF1 extract. With regard to the analysis of this extract, it revealed a signal with mass 343.156 Da (safficinolide) that was not detected in any other extract. Other common features observed in the three supercritical antisolvent fractionation extracts were the higher enrichment in 11,12-dimethylrosmanol and the lower rosmanol peak area compared to SFE1 and SFE2, suggesting that this latter diterpene is better extracted using SC-CO_2_ and SC-CO_2_ with ethanol as solvents. To note, betulinic, oleanolic and ursolic acids show identical molecular formula, and thus, the same *m*/*z*; however, they were tentatively identified by their retention times according to a similar separation carried out by Kontogianni et al. (2013) [19]. The chromatographic profile of SAF3 extract analysis showed maximum peak areas for signals at *m*/*z* 455.352 that may correspond to ursolic acid (455.352 Da), a compound with demonstrated cytotoxic activity [19], and four other masses that correspond to less polar compounds (467.317, 467.318, 615.406, and 551.374 Da).

Besides the low CA and CS content in PLE sample, this extract showed a distinctive chromatographic profile compared to the rest of the extracts. For instance, peak signals with masses corresponding to syringic acid, gallocathechin, rosmarinic acid and other two non-identified compounds (NI1 and NI2) were exclusively detected in the analysis of PLE extract. The distinctive presence of these phenolic acids and flavonoid evidences a higher selectivity for polar compounds of the PLE process compared to the other extraction procedures. Furthermore, the analysis of PLE extract also revealed that betulinic acid was only detected in this extract. Interestingly, Kontogianni et al. (2013) attributed part of the cytotoxic activity observed in a CA-enriched rosemary extract obtained using solid-liquid extraction to the presence of the triterpenoids betulinic and ursolic acids in addition to CA [19]. More recently, in a study based on the fractionation of a rosemary extract obtained by SFE, CA was the major contributor to the anti-proliferative activity, followed by CS and also betulinic acid [58]. In that case, betulinic acid concentration in the extract was 2.1 µM, and the incubation of HT-29 cells with a purified fraction from the same extract containing 81% betulinic acid and 19% hinokione showed the same cytotoxicity as the whole extract. In another published work, Rzeski et al. (2006) demonstrated that betulinic acid acts as an effective anticancer agent by inducing growth arrest and apoptosis in concentration-dependent manner, with HT-29 cells being particularly sensitive to this pentacyclic triterpenoid [60]. However, reported IC50 values for betulinic acid by different research groups for the same cell model (HT-29) are discordant (2.7 µM, [60]; 13.9 µM, [61]; and 32.7 µM, [62]). The extremely low aqueous solubility (<1 µM), high protein binding (>99%) and poor membrane permeability of this compound [63,64] could explain the lack of robustness in data obtained under slightly different culturing conditions. Interestingly, our findings indicate that betulinic acid was only present in the less active extract, suggesting that this compound is not among the most relevant active constituents in the rosemary extracts obtained in the present study. To gain further insight into the chemical differences among the extracts, the main volatile compounds were identified and quantified by GC-MS analysis.

In all cases, 1,8-cineole and camphor were the two more abundant volatile compounds found in the GC chromatograms profiles. These results are in good agreement with others reported in the literature for supercritical fluids and hydrodistillated rosemary extracts [14,44,65]. As it is shown in Figure 2B, quantitative data indicated that 1,8-cineole and camphor were present at levels ranging from 6.8 to 46.4 and from 8.6 to 50.3 mg/g extract, respectively. As a general trend, both monoterpenes were more abundant in the more active extracts. Single-step and two-step sequential extraction procedures seemed to be more selective for 1,8-cineole than camphor; the concentration of both volatile monoterpenoids was lower in SFE2 (approximately 25% and 37% lower). This can be explained by the removal of most aromatic and highly volatile components (mono- and sesquiterpenes, and their oxygenated derivates) in the two-step sequential SFE process, leading to a more volatile-free extract (SFE2). The opposite behavior occurred with the antisolvent fractionation methodology (SAF) where extracts showed higher camphor content, which is less volatile than 1,8-cineole.

The cytotoxic activity of this latter compound has been demonstrated to be within the low millimolar range against human colorectal HCT116 cells [66], which is well above (e.g., in 30 µg extract/mL, the concentration of 1,8-cineole ranged from 1.32 to 9.03 µM) its concentration in the rosemary extracts of our present study. A similar potency has been recently reported for camphor monoterpene, exerting a 50% reduction in viability at concentrations of 5.5 and 4.5 mM, respectively in HT-29 and HCT116 cells [67]. These published data suggest that both monoterpenes, when individually assayed, have only modest cytotoxic activity compared to other compounds in the rosemary extracts. According to our quantitative data, their content in the extracts seems to be below their reported inhibitory concentrations. In spite of that, camphor and 1,8 cineole were, with respect to PLE extract, enriched more than three-fold in the rest of the extracts with a comparably good activity. This is in accordance with findings by other groups [55], indicating that the contribution of volatile monoterpenes to the anti-proliferative activity of the extracts, particularly those with high CA content, cannot be totally dismissed.

## 3. Materials and Methods

### 3.1. Samples and Reagents

Raw material consisted of rosemary (*Rosmarinus officinalis* L.) dried leaves obtained from Herboristeria Murciana (Murcia, Spain) that were ground at low temperature (by its mixing with small rocks of dry ice) employing a knife mill (Grindomix GM200, Retsch GmbH, Haan, Germany). Sample particle size was in the range of 500 and 900 µm. Next, grinded samples were vacuum packed and stored at 4 °C until further use. Carbon dioxide (99% purity) purchased from Carburos Metálicos (X50S, Barcelona, Spain) was employed for the anti-solvent fractionation and supercritical fluids extractions. Ethanol (99.5%), provided by VWR Chemicals (Fontenay-sous-Bois, France), and ultrapure water, obtained from a Millipore system (Billerica, MA, USA), were used for PLE. Phenols standards such as rosmarinic acid (RA, ≥98%), CA (≥97%), CS (≥98%), gallic acid, as well as other chemicals as 1,8-cineole (99%), camphor (95%), 6-hydroxy-2,5,7,8-tetramethylchroman-2-carboxylic acid (Trolox, ≥97%), ABTS (≥99%), DPPH (99%) were purchased from Sigma-Aldrich (Madrid, Spain). Folin-Ciocalteu phenol reagent (2N) was provided by Merck (Darmstadt, Germany). For the UHPLC-qTOF-MS analyses, MS grade ACN and water from LabScan (Dublin, Ireland) were employed. Dry extracts were dissolved in DMSO (Sigma-Aldrich) at the appropriate concentrations and stored as aliquots at −80 °C until their use in cell proliferation inhibition assays.

### 3.2. Rosemary-Leaf Extraction Procedures

The extraction procedures used in the present work to obtain the different rosemary extracts are described in this section (main extraction parameters are also summarized in Table 1).

#### 3.2.1. Pressurized Liquid Extraction (PLE)

Hydroalcoholic rosemary extracts were obtained using an accelerated solvent extractor (ASE 200, Dionex, Sunnyvale, CA, USA), equipped with a solvent controller unit. The extraction protocol has been described in depth previously [41]. Briefly, a mixture of ethanol + water (80:20, *v*/*v* or 76:24 *w*/*w*) was employed as solvent at 100 bar and 150 °C for 20 min. Successive extractions were performed until complete 1000 mL of total extract. An aliquot was submitted to rotary evaporation and freeze-drying (Labconco Corporation, Kansas City, MO, USA) to eliminate the ethanol and water, respectively, thus obtaining the dry extract. The remaining liquid extract was stored with N_2_ atmosphere in the dark at −20 °C until their use for the antisolvent fractionation experiments.

#### 3.2.2. Single-Step SFE Process

Single-step SFE process was performed as previously reported in detail by Herrero et al. 2010 [43]. Extraction was carried out in triplicate in a pilot scale supercritical fluid extractor (model SF2000, Thar Technology, Pittsburgh, PA, USA) including a 2 L cylinder extraction cell and two different separators with independent control of temperature and pressure. The extraction conditions employed for the single-step SFE process were 150 bar, 40 °C for 300 min, as described in Table 1. CO_2_ flow rate was set to 60 g·min^−1^ and 7% ethanol was employed as co-solvent. After the extractions, ethanol was eliminated by rotary evaporation (R-210, Büchi Labortechnik AG, Flawil, Switzerland). The extracts were stored with N_2_ atmosphere in the dark at −20 °C.

#### 3.2.3. Two-Step Sequential SFE

The same equipment employed for the single-step SFE process was used for the two-sequential SFE extraction as described by Sánchez-Camargo et al. (2014) [42]. The extraction protocol involved two consecutive steps with the following conditions: (1) 300 bar for 60 min and (2) 150 bar, 7% ethanol (*w*/*w*) for 120 min. Both stages were performed at 40 °C and using 60 g·min^−1^ of CO_2_ flow. Once the extracts were obtained in triplicate, ethanol was also eliminated by rotary evaporation. Dried extracts were kept at −20 °C, in the dark and in N_2_ storage atmosphere.

#### 3.2.4. Supercritical Antisolvent Fractionation (SAF) at Pilot Scale

**Figure 3:**
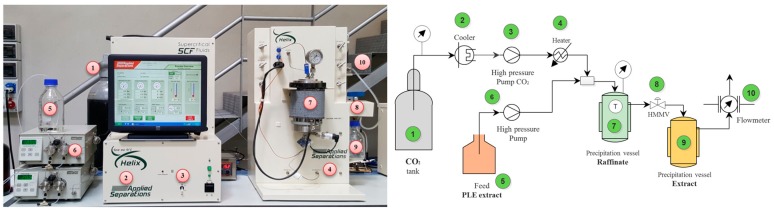
Scheme of the supercritical antisolvent fractionation (SAF) pilot scale system used in this work.

A semi-continuous SAF was carried out in a Spe-ed Helix™ supercritical fluid extractor from Applied Separations (Allentown, PA, USA). Originally, this equipment was designed to carry out SFE (with or without a co-solvent) and subcritical water extraction (SWE); in the present work, some modifications were introduced in the system in order to use it as a SAF at pilot scale (Figure 1). Owing to the unavailability of a pilot-scale PLE equipment and in order to obtain enough volume of PLE extract to feed SAF processes, PLE extract was made of a pool of extracts obtained in various independent extractions using lab-scale PLE equipment. Then, based on previous lab scale results [41] three different extraction conditions (see Table 1) were selected to be compared, and a 60-fold scale up from bench to pilot scale was achieved. After the PLE process, the resulting hydroalcoholic rosemary extract was properly diluted to obtain the extracts with 44.2% and 76.0% (*w*/*w*) of ethanol (or 50% and 80% (*v*/*v*) ethanol, respectively), for SAF1-2 and SAF3, respectively and according to the previously optimized conditions. Then, diluted extracts were filtered through Whatman cellulose filter paper and 100 mL samples were employed for each fractionation, accomplished in triplicate. In the Figure 3 is showed an scheme of the SAF pilot scale equipment employed. Briefly, CO_2_ was provided from a pressurized cylinder (1), subcooled in a chiller (2) and then conducted to a high pressure pump (3). Immediately, the CO_2_ was compressed at 100 bar and continuously pumped at a flow of 10 L·min^−1^ adjusted by a heated micrometering valve (HMMV) (8) at the exit of the system through of CO_2_ mass flowmeter (10). At the same time, PLE extract (5) was fed through another high pressure pump at suitable flow according to feed/SC-CO_2_ rate (6). In a T-tube device, the CO_2_ (4) and the feed (rosemary extract) was mixed before reaching the raffinate chamber separation. A polypropylene vessel (250 mL) was placed inside the high-pressure stainless-steel extraction cell to act as precipitation raffinate vessel (7). The temperature of the raffinate separation chamber was kept at 40 °C by a heating jacket and measured by an internal thermocouple. Once the aqueous fraction was separated, the fraction soluble in SC–CO_2_ + ethanol was precipitated in a glass bottle (9) acting as extract chamber separation, which was kept at room temperature (25 °C). During the SAF time, a whole process overview was carried out through to a software and screen coupled to the system. After obtaining the extract fraction, the ethanol was removed by rotary evaporation and the water was eliminated by freeze-drying (Labconco Corporation, Kansas City, MO, USA). Dried extracts were kept under freezing at −20 °C N_2_ atmosphere until analysis. The extraction yield (or recovery) was determined gravimetrically, as the ratio of the mass of dry extract recovered in the separators and the mass of dry PLE extract fed, and expressed as a percentage.

### 3.3. Chemical Characterization of Rosemary Extracts

#### 3.3.1. Liquid Chromatography-Diode Array Detection-Mass Spectrometry

Major phenolic compounds typically present in rosemary extracts (CA and CS) were quantified employing an ACCELA UHPLC system (Thermo Scientific™, San Jose, CA, USA) coupled to a TSQ Quantum (Thermo Scientific™) triple quadrupole analyzer via an electrospray interface. Briefly, the analytical conditions employed consisted on the use of a Hypersil Gold column (50 mm × 2.1 mm, d.p. 1.9 μm) (Thermo Scientific™) using as mobile phases 0.1% formic acid in acetonitrile (A) and 0.1% formic acid in water (B) eluted according to the following gradient: 0 min, 95% B; 0.35 min, 95% B; 3.5 min, 40% B; 6.2 min, 5% B; 7 min, 95% B; 10 min, 95% B. The flow rate was 0.4 mL/min and the injection volume was 5 μL. The diode array detector recorded the spectra from 200 to 450 nm. Calibration curves were constructed using external calibration method to quantify CA and CS (0.0625–2.0 µg/mL and 0.313–5.0 µg/mL, respectively). The mass spectrometer was operated in the negative electrospray ionization (ESI) mode using multiple reaction monitoring (MRM) with a Q1 and Q3 resolution of 0.7 Da FWHM, scan width 0.010 Da and scan time of 0.10 s. The values corresponding to the tube lens voltage and collision energy for each ion transition were optimized for each quantified compound: carnosic acid *m*/*z* 331.14 ([M-H]^−^) and *m*/*z* 287.19 ([M-H]^−^, product ion using 62 V and 26 V as tube lens value (TLV) and CE, respectively), and for carnosol *m*/*z* 329.16 ([M-H]^−^) and 285.15 *m*/*z* ([M-H]^−^, product ion using 45 V and 20 V as TLV and CE, respectively).

#### 3.3.2. Liquid Chromatography-Quadrupole Time-Of-Flight Mass Spectrometry (LC-Q/TOF-MS)

To obtain a more complete chemical characterization of the rosemary extracts, these were analyzed by liquid chromatography coupled to a high-resolution mass spectrometer. It consisted of an ultrahigh performance liquid chromatography (UHPLC) system 1290 from Agilent (Agilent Technologies, Santa Clara, CA, USA) coupled to a quadrupole-time-of-flight mass spectrometer (Q/TOF MS) Agilent 6540 that was equipped with an orthogonal ESI source (Agilent Jet Stream, AJS, Santa Clara, CA, USA), and controlled by a PC running the Mass Hunter Workstation software 4.0 (MH) from Agilent. The analyses were performed in negative ion mode. Chromatographic separation of the extracts was achieved using a Hypersil Gold column (50 mm × 2.1 mm, d.p. 1.9 μm) (Thermo Scientific™) with a mobile phase composition of acetonitrile (+0.1% formic acid, A) and water (+0.1% formic acid, B). The gradient program was as follows: 0 min, 95% B; 0.35 min, 95% B; 3.5 min, 30% B; 6.2 min, 5% B; 9 min, 95% B. The flow rate was 0.4 mL/min with an injection volume of 2 µL. The diode array detector recorded the spectra from 200 to 450 nm. The extracts were injected to a concentration of 50 µg/mL. MS parameters were the following: capillary voltage, 4000 V; nebulizer pressure, 30 psi; drying gas flow rate, 10 L/min; gas temperature, 300 °C; skimmer voltage, 45 V; fragmentor voltage, 125 V. The QTOF-MS was set to acquire *m*/*z* ranging between 50 and 1100 amu at a scan rate of 5 spectra per s. External calibration of the TOF MS was carried out using a commercial mixture from Agilent with the following *m*/*z* values: 301.9981, 601.9790, 1033.9881, 1333.9689, 1633.9498, 1933.9306, 2233.9115, 2533.8923 and 2833.8731. The identification of CA and CS was based on the standard samples. The other compounds were tentatively identified in accordance with the molecular formula and the exact mass of the compound.

#### 3.3.3. Gas Chromatography-Mass Spectrometry (GC-MS)

The main volatile compounds (camphor and 1,8-cineole) in rosemary extracts were quantified using the GC-MS method developed by Sánchez-Camargo et al. (2014) [42] with some modifications. A GCMS-QP2010 plus system (Shimadzu, Kyoto, Japan) equipped with a DB-5ms column (30 m × 0.25 mm I.D. × 0.25 µm df, Quadrex Corporation, Woodbridge, CT, USA) was used. The oven temperature program for the separation was carried out as follow: 60 °C, held for 4 min, and then raised to 100 °C at 3 °C/min followed by an increase to 110 °C at 1 °C/min, and then to 150 °C at 5 °C/min. Finally, the temperature was raised to 300 °C at 15 °C/min and this value was held for 25 min. Injection volume was 1.6 µL in split mode (split ratio 1:10) maintaining an injector temperature of 250 °C. The carrier gas employed was He at 36.4 cm·s^−1^. MS detection parameters were: interface and source temperatures were 280 °C and 230 °C, respectively; mass range, *m*/*z* 40–500; scan speed: 2500 amu/s; event time 0.20 s. Collection and handling of data was performed using the GCMS solution (ver. 2.50 SU3, Shimadzu) software. A commercial mass spectral database (Wiley) and the linear retention indices (LRI) of the resolved peaks were used to identify the different compounds. For the determination of LRIs, a hydrocarbon mixture ranging from C_8_ to C_30_ (Hydrocarbons/C5–C30, straight-chain alkanes, Sigma-Aldrich) was employed and analyzed under the same experimental conditions as the sample. After identification, calibration curves of camphor and 1,8-cineole were employed to quantify their content in the sample (2.5–25.0 µg/mL, for both compounds). GC-MS analyses were carried out in triplicate.

### 3.4. Total Phenols Content (Folin-Ciocateu Method)

The quantification of total phenols content (TPC) in the rosemary extracts was carried out using the Folin–Ciocalteu method with some modifications [68]. Briefly, 600 µL of water were mixed with 10 µL of each extracts (2.5–5 mg/mL of rosemary extract in ethanol or ethanol: water mixtures) to which 50 µL of undiluted Folin–Ciocalteu reagent (2N) was subsequently added. After 1 min, 150 µL of 20% (*w*/*v*) Na_2_CO_3_ were added and the volume was made up to 1.0 mL with water. After 2 h of incubation at 25 °C, 300 µL of the mixture was transferred into a well of a 96-well microplate. The absorbance was measured at 760 nm in a microplate spectrophotometer reader (Synergy HT, Bio Tek Instruments, Winooski, VT, USA). A gallic acid calibration curve (0.032–2.00 mg/mL) was elaborated in the same way and the TPC was expressed as mg of gallic acid (GAE) per g of extract. All analyses were done in triplicate.

### 3.5. Antioxidant Activity In Vitro Assays

#### 3.5.1. DPPH Radical Scavenging Assay

The antioxidant activity was determined following the adjusted procedure described by Brand-Williams, Cuvelier and Berset (1995) [69] employing 1,1-diphenyl-2-picrilhidrazyl (DPPH) reagent. A stock solution was prepared dissolving 23.5 mg of DPPH in 100 mL of methanol which was further diluted 1:10 with methanol to give the working solution. Both stock and working solutions were stored at 4 °C until use. A volume of 975 μL of DPPH diluted solution was added to 25 μL of each extract concentration solution and the reaction was kept at darkness for 4 h at room temperature. Different concentrations (from 0.0625 to 0.5 mg/mL) of each extract were tested. Once the reaction was finished, 300 μL of this mixture was transferred into a well of a microplate, and the absorbance was measured at 516 nm in a microplate spectrophotometer reader (Synergy HT). DPPH–methanol solution was used as a reference sample. The DPPH concentration remaining in the reaction medium was calculated from a calibration curve. The extract concentration (expressed in µg/mL) responsible for a 50% decrease in the initial activity of the DPPH (EC_50_, μg/mL) was calculated by linear regression of the percentage remaining DPPH curve obtained for all the extract concentrations. Therefore, the lower the EC_50_ value, the higher the antioxidant capacity. Measurements were done in triplicate.

#### 3.5.2. TEAC Assay

The antioxidant capacity of the different rosemary extracts was determined by TEAC assay following the ABTS radical method based on the procedure described by Re et al. (1999) with some modifications [70]. ABTS^•+^ radical was produced by reacting 7 mM ABTS and 2.45 mM potassium persulfate in the dark at room temperature during 16 h before its use. The aqueous ABTS^•+^ solution was diluted with 5 mM phosphate buffer (pH 7.4) until achieve an absorbance of 0.7 (±0.02) at 734 nm. One mL of ABTS^•+^ solution was mixed with 10 microliters of sample (five different concentrations) in a 1.5-mL vial and 300 µL of the mixture were transferred into a 96-well microplate. The absorbance was measured at 734 nm every 5 min during 45 min in a microplate spectrophotometer reader (Synergy HT). Trolox was used as a reference standard and results were expressed as TEAC values (mmol of Trolox/g extract). These values were obtained from five different concentrations of each extract tested (between 0.0625–1 mg/mL) in the assay giving a linear response between 20% and 80% blank absorbance. All analyses were done in triplicate.

### 3.6. Cell Culture

Colon adenocarcinoma HT-29 and HCT116 cell lines were purchased from ATCC (American Type Culture Collection, LGC Promochem, UK). Cells were cultured in McCoy’s 5A supplemented with 10% (*v*/*v*) heat inactivated FBS (Fetal Bovine Serum), 50 U/mL streptomycin, and 50 U/mL penicillin G, in humidified atmosphere and 5% CO_2_ at 37 °C. Cells were trypsinized when reached ~50% confluence, neutralized with culture medium, seeded at 5000 cells/well, and allowed to adhere overnight at 37 °C. MTT (3-(4,5-dimethylthiazol-2-yl)-2,5-diphenyltetrazolium bromide) assay method was used to assess the antiproliferative activity of the extracts. Briefly, cells were incubated with the vehicle (0.2% (*v*/*v*) DMSO) regarded as untreated controls or with different extract concentrations and incubated for different times (24 and 72 h). After incubations, cells were incubated with MTT solution (0.5 mg/mL) at 37 °C for 3 h. Then, the medium was aspirated, and the purple formazan crystals were dissolved in DMSO. The absorbance at 570 nm was measured in a microplate reader (Synergy HT). Based on the NIH definitions [71], the percentage of growth (PG) was calculated with the formula PG = 100 [(T − T_0_)/(C − T_0_)] when T ≥ T_0_, or PG = 100 [(T − T_0_)/C] when T < T_0_, T being the optical density of treated cells, C the optical density of control cells, and T_0_ the optical density at time zero. Then, PG values were used to calculate the parameters related with cell proliferation after 24 h of treatment (GI_50_, 50% growth inhibition; and LC_50_, 50% lethal concentration) using SigmaPlot v12.5 software (Systat Software Inc., Erkrath, Germany). The results are provided as the mean ± SEM of at least three independent experiments, each performed in triplicate.

### 3.7. Statistical Analysis

Data analysis were carried out using the software Statgraphics Centurion XVI^®^ (StatPoint Technologies, Inc., Warrenton, VA, USA) using a level of significance set at 95%. One-way analysis of variance (ANOVA), together with *F*-test, was employed to group extracts, based on statistically significant differences. Mean values were compared using Student-Newman-Keuls multiple comparison procedure and differences were considered statistically significant if *p* < 0.05.

## 4. Conclusions

Taken together, our data highlight the good potential of novel green extraction strategies for obtaining rosemary extracts with potent inhibitory activity of cancer cell proliferation. The most active rosemary extracts were characterized by containing CA and CS at concentrations above 263.7 and 33.9 mg/g extract, respectively. However, above those concentrations, a lack of positive correlation between the extract potency and the CA and CS content was observed, which suggest the potential presence of other unidentified constituents that may also contribute to the observed anti-proliferative effect of the rosemary extracts. The extracts obtained using an integrated process that involved PLE and SAF provided the most active rosemary extracts against both colon cancer cell lines. The compounds tentatively identified as rosmaridiphenol and safficinolide were exclusively identified in SAF1-3 and SAF1 extracts, respectively, suggesting that they are possible additional contributors to the observed strong anti-proliferative activity of CA and CS in SAF extracts. Contrasting with published data that suggest betulinic acid as a relevant contributor to the anti-proliferative activity of rosemary extracts, our data indicate this compound is not among the most active constituents in the rosemary extracts obtained in the present work. In addition, although the concentrations of the two major monoterpenes in the extracts are well below their reported active concentration in the same cell models, their potential synergistic contributions to the anti-proliferative activity of the extracts should not be dismissed. This study also illustrates the complexity of assigning the anti-proliferative activity to individual or group of compounds in complex extracts when more than one compound is active. This challenging task is crucial for the development of extraction processes that provide optimal content of active compounds.

## Figures and Tables

**Table 1:** Experimental conditions employed for the extraction of the different rosemary extracts.

Process	Sample Name	Pressure (Bar)	Temperature (°C)	Feed/SC-CO_2_ Ratio	%H_2_O (*w*/*w*)	%Ethanol (*w*/*w*)	Process Time (min)
PLE	PLE	100	150	-	24.0	76.0 **	20
Single-step SFE	SFE1	150	40	-	-	7.0 *	300
Two-step SFE	SFE2	300	40	-	-	0	60
		150	40	-	-	7.0 *	120
SAF	SAF1	100	40	0.025	55.8	44.2 **	180
SAF	SAF2	100	40	0.100	55.8	44.2 **	60
SAF	SAF3	100	40	0.025	24.0	76.0 **	180

*: Ethanol as co-solvent; **: ethanol in the solvent mixture. SC-CO_2_: Supercritical carbon dioxide; PLE: Pressurized liquid extraction; SAF: Supercritical antisolvent fractionation; SFE: Supercritical fluid extraction.

**Table 2:** Extraction yield (% dry weight), total phenolic content (TPC) and antioxidant activity (Trolox equivalent antioxidant capacity (TEAC) and 1,1-diphenyl-2-picrilhidrazyl (DPPH) assays) obtained for the different extracts.

Sample	Yield (g/100 g Sample)	TPC ^1^	TEAC ^2^	EC_50_^3^
PLE	38.46 ^d^ ± 1.99	233.88 ^f^ ± 4.43	2.75 ^b^ ± 0.04	7.70 ^d^ ± 0.33
SFE1	6.74 ^a^ ± 0.33	134.42 ^a^ ± 4.51	1.87 ^a^ ± 0.05	7.03 ^e^ ± 0.15
SFE2	4.68 ^a^ ± 0.01	169.01 ^b^ ± 8.61	2.64 ^b^ ± 0.03	5.61 ^c^ ± 0.11
SAF1	20.65 ^c^ ± 1.74	220.05 ^e^ ± 5.82	4.09 ^d^ ± 0.15	3.39 ^a^ ± 0.08
SAF2	5.74 ^a^ ± 0.45	203.04 ^d^ ± 7.81	3.67 ^c^ ± 0.09	4.12 ^b^ ± 0.12
SAF3	15.36 ^b^ ± 1.41	188.55 ^c^ ± 1.06	3.80 ^c^ ± 0.20	7.83 ^d^ ± 0.22

In each column, superscripts letters mean groups not statistically different (*p* > 0.05), as analyzed by one-way ANOVA. ^1^ mg gallic acid equivalents (GAE) g^−1^ extract; ^2^ mmol trolox equivalents g^−1^ extract; ^3^ Efficient concentrations, µg extract mL^−1^ obtained by DPPH assay.

**Table 3:** Tentatively identification by Liquid Chromatography-Quadrupole Time-of-Flight Mass Spectrometry (LC-Q/TOF-MS) of compounds present in rosemary leaf extracts.

Peak	Rt (min)	[H-M]^−^	Molecular Formula	Identification	Peak Area (Mean ± SD) (× 10^6^)					
					PLE	SFE1	SFE2	SAF1	SAF2	SAF3
1	0.479	387.1171	C_13_ H_24_ O_13_	NI1	3.91 ± 0.24	-	-	-	-	-
2	0.994	198.0528	C_9_ H_10_ O_5_	Siringic acid	0.36 ± 0.02	-	-	-	-	-
3	1.737	306.0798	C_15_ H_14_ O_7_	Gallocathechin	0.67 ± 0.04	-	-	-	-	-
4	1.797	387.1678	C_18_ H_28_ O_9_	NI2	0.99 ± 0.03	-	-	-	-	-
5	2.274	360.0845	C_18_ H_16_ O_8_	Rosmarinic acid	4.10 ± 0.12	-	-	-	-	-
6	2.730	207.0636	C_11_ H_12_ O_4_	Tryhydroxycinnamic acid derivate	-	-	-	1.024 ^c^ ± 0.006	0.71 ^b^ ± 0.01	0.61 ^a^ ± 0.05
7	3.178	345.1737	C_20_ H_26_ O_5_	Rosmanol	0.930 ^a^ ± 0.009	6.16 ^f^ ± 0.42	4.80 ^e^ ± 0.15	3.90 ^d^ ± 0.02	2.37 ^b^ ± 0.01	2.87 ^c^ ± 0.11
8	3.275	345.1706	C_20_ H_26_ O_5_	Epirosmanol/Isorosmanol	-	1.72 ^b^ ± 0.06	1.53 ^a^ ± 0.01	2.68 ^c^ ± 0.15	1.80 ^b^ ± 0.007	1.69 ^a,b^ ± 0.07
9	3.392	283.0617	C_16_ H_12_ O_5_	Genkwanin	0.78 ^a^ ± 0.05	2.29 ^d^ ± 0.02	1.79 ^b^ ± 0.01	2.02 ^c^ ± 0.01	1.88 ^b^ ± 0.02	1.90 ^b,c^ ± 0.06
10	3.843	343.1563	C_20_ H_24_ O_5_	Safficinolide	-	-	-	0.57 ± 0.01	-	-
11	3.883	331.1562	C_19_ H_24_ O_5_	NI3	-	-	-	2.06 ^c^ ± 0.09	1.26 ^b^ ± 0.02	0.99 ^a^ ± 0.05
12	3.960	329.1754	C_20_ H_26_ O_4_	Carnosol	3.76 ^a^ ± 0.12	15.49 ^b^ ± 0.17	30.90 ^e^ ± 0.90	29.76 ^e^ ± 0.88	22.63 ^d^ ± 0.07	18.84 ^c^ ± 0.16
13	4.111	343.1630	C_20_ H_24_ O_5_	Rosmadial	-	0.92 ^b^ ± 0.06	-	0.99 ^c^ ± 0.01	0.91 ^b^ ± 0.01	0.708 ^a^ ± 0.002
14	4.158	373.2037	C_22_ H_30_ O_5_	11,12-Dimethylrosmanol	-	-	2.17 ^a^ ± 0.14	4.53 ^c^ ± 0.23	3.22 ^b^ ± 0.03	3.26 ^b^ ± 0.02
15	4.258	325.1865	C_21_ H_26_ O_3_	NI4	1.39 ^a^ ± 0.06	2.29 ^c,d^ ± 0.25	1.86 ^b^ ± 0.14	1.96 ^b,c^ ± 0.24	2.51 ^d^ ± 0.03	1.37 ^c^ ± 0.08
16	4.338	331.1952	C_20_ H_28_ O_4_	Carnosic Acid	21.80 ^a^ ± 1.04	42.86 ^b^ ± 0.36	50.78 ^d^ ± 2.44	49.28 ^d^ ± 1.09	50.01 ^d^ ± 0.25	45.95 ^c^ ± 0.53
17	4.575	345.2133	C_21_ H_30_ O_4_	Methyl carnosate/12-methoxy-carnosic acid	1.50 ^a^ ± 0.04	8.04 ^b^ ± 0.25	12.63 ^d^ ± 0.65	12.42 ^d^ ± 0.89	12.33 ^d^ ± 0.14	9.95 ^c^ ± 0.43
18	4.753	315.1965	C_20_ H_28_ O_3_	Rosmaridiphenol	-	-	-	0.50 ^b^ ± 0.02	0.509 ^b^ ± 0.008	0.21 ^a^ ±0.02
19	4.799	317.2107	C_20_ H_30_ O_3_	NI5	-	2.27 ^a,b^ ± 0.07	2.47 ^b^ ± 0.14	2.15 ^a^ ± 0.08	2.138 ^a^ ± 0.006	2.40 ^b^ ± 0.11
20	4.818	455.3422	C_26_ H_48_ O_6_	Betulinic Acid *	2.304 ± 0.003	-	-	-	-	-
21	4.976	455.3650	C_30_ H_48_ O_3_	Oleanolic acid *	7.99 ^c^ ± 0.52	5.56 ^b^ ± 0.22	5.15 ^b^ ± 0.08	4.08 ^a^ ± 0.14	5.25 ^b^ ± 0.03	5.14 ^b^ ± 0.32
22	5.110	455.3515	C_30_ H_48_ O_3_	Ursolic acid *	-	1.84 ^c^ ± 0.19	1.51 ^b^ ± 0.05	1.368 ^a^ ± 0.009	1.542 ^b^ ± 0.007	2.24 ^d^ ± 0.02
23	5.441	479.2785	C_30_ H_40_ O_5_	NI6	-	1.07 ^d^ ± 0.02	0.60 ^a^ ± 0.04	0.94 ^c^ ± 0.07	0.759 ^b^ ± 0.007	1.15 ^d^ ± 0.08
24	5.621	331.1921	C_20_ H_28_ O_4_	NI7	-	0.12 ^a^ ± 0.02	1.61 ^d^ ± 0.10	1.89 ^e^ ± 0.01	0.521 ^c^ ± 0.004	0.33 ^b^ ± 0.03
25	6.414	467.3168	C_30_ H_44_ O_4_	NI8	1.23 ^a^ ± 0.01	4.42 ^d^ ± 0.16	4.99 ^e^ ± 0.31	1.63 ^b,c^ ± 0.04	1.79 ^c^ ± 0.03	5.48 ^f^ ± 0.02
26	6.658	467.3184	C_30_ H_44_ O_4_	NI9	0.83 ^a^ ± 0.06	3.32 ^c^ ± 0.11	2.38 ^b^ ± 0.08	1.06 ^d^ ± 0.03	1.03 ^a^ ± 0.02	4.48 ^d^ ± 0.17
27	6.840	615.4061	C_33_ H_60_ O_10_	NI10	0.075 ^a^ ± 0.018	0.50 ^c^ ± 0.04	0.33 ^b^ ± 0.02	-	-	0.57 ^d^ ± 0.03
28	6.997	551.3749	C_35_ H_52_ O_5_	NI11	-	0.305 ^b^ ± 0.005	0.20 ^a^ ± 0.02	-	-	0.46 ^c^ ± 0.03
29	7.290	535.3794	C_35_ H_52_ O_4_	NI12	-	-	0.34 ± 0.02	-	-	-

a–f, for each peak (row), peak area mean values that do not share subscripts differ by *p* < 0.05 as analyzed by one-way ANOVA; * The order of these compounds is suggested according to the identification performed by Kontogianni et al., 2013 [19].

## Supplementary Materials

Supplementary materials can be found at www.mdpi.com/1422-0067/17/12/2046/s1.
