# Supplementary material for: Comparative Study of Green Sub- and Supercritical Processes to Obtain Carnosic Acid and Carnosol-Enriched Rosemary Extracts with in Vitro Anti-Proliferative Activity on Colon Cancer Cells
Source: Int J Mol Sci. 2016 Dec 7;17(12):2046. doi: 10.3390/ijms17122046 (PMC5187846; doi:10.3390/ijms17122046)
Supplement: Supplementary file 1 [file ijms-17-02046-s001.pdf]

Supplementary Materials: Comparative Study of Green Sub- and Supercritical Processes to Obtain Carnosic Acid and Carnosol-Enriched Rosemary Extracts with In Vitro Anti-Proliferative Activity on Colon Cancer Cells

Andrea del Pilar Sánchez-Camargo, Virginia García-Cañas, Miguel Herrero, Alejandro Cifuentes and Elena Ibáñez

Table S1. Quantification of main phenolic and volatiles compounds in the rosemary extracts. Carnosic acid (CA), Carnosol (CS). Concentrations indicated as mg/g extract ± SD. Values are the mean of three replicates.

Sample	mg/g Extract				
	CA	CS	CA + CS	1.8-Cineole	Camphor
PLE	104.96 ± 1.87 ^{c}	10.66 ± 0.06 ^{c}	115.63 ± 1.93 ^{c}	6.78 ± 0.14 ^{c}	8.57 ± 0.43 ^{c}
SFE1	355.73 ± 17.44 ^{a}	39.23 ± 1.91 ^{a}	394.97 ± 17.91 ^{a}	46.42 ± 1.95 ^{a}	31.97 ± 1.90 ^{a}
SFE2	443.28 ± 4.09 ^{b}	88.88 ± 5.41 ^{b}	532.16 ± 4.73 ^{b}	34.88 ± 1.30 ^{b}	20.23 ± 0.82 ^{b}
SAF1	386.49 ± 1.98 ^{d}	64.24 ± 4.04 ^{d}	450.73 ± 4.70 ^{d}	18.11 ± 0.33 ^{d}	50.02 ± 2.62 ^{d}
SAF2	354.92 ± 7.63 ^{a}	44.04 ± 0.73 ^{a}	398.96 ± 8.00 ^{a}	36.79 ± 1.76 ^{b}	50.34 ± 2.93 ^{d}
SAF3	263.70 ± 8.07 ^{e}	33.89 ± 0.51 ^{e}	297.59 ± 8.57 ^{e}	22.38 ± 0.89 ^{e}	31.38 ± 1.29 ^{a}

PLE, Pressurized liquid extraction; SFE, Supercritical fluid extraction; SAF, Supercritical antisolvent fractionation. In each column, superscripts mean groups not statistically different ($p > 0.05$), as analyzed by one-way ANOVA.

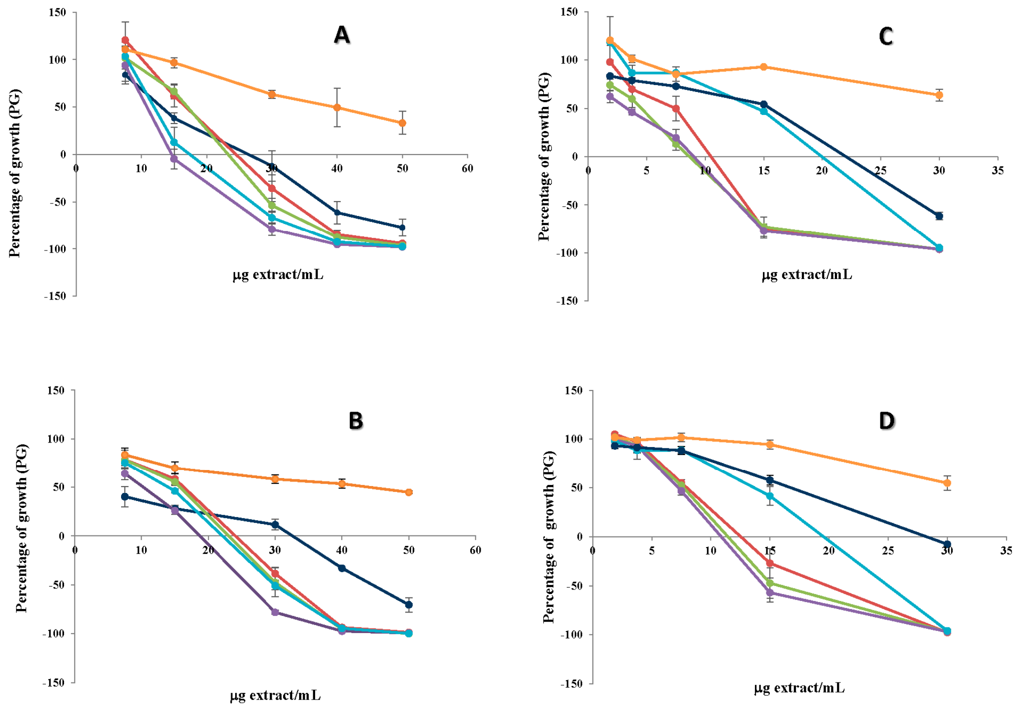

Figure S1. Percentage of growth (PG) of HT-29 and HCT116 colon cancer cells exposed to the different extracts concentrations at different exposure times. Calculated values for HT-29 at 24 h (**A**) and 72 h (**B**); and for HCT116 at 24 h (**C**) and 72 h (**D**). SFE1 (blue line), SFE2 (cyan line), PLE (orange line), SAF1 (red line), SAF2 (green line), SAF3 (violet line). Error bars represent standard error of the mean (SEM).

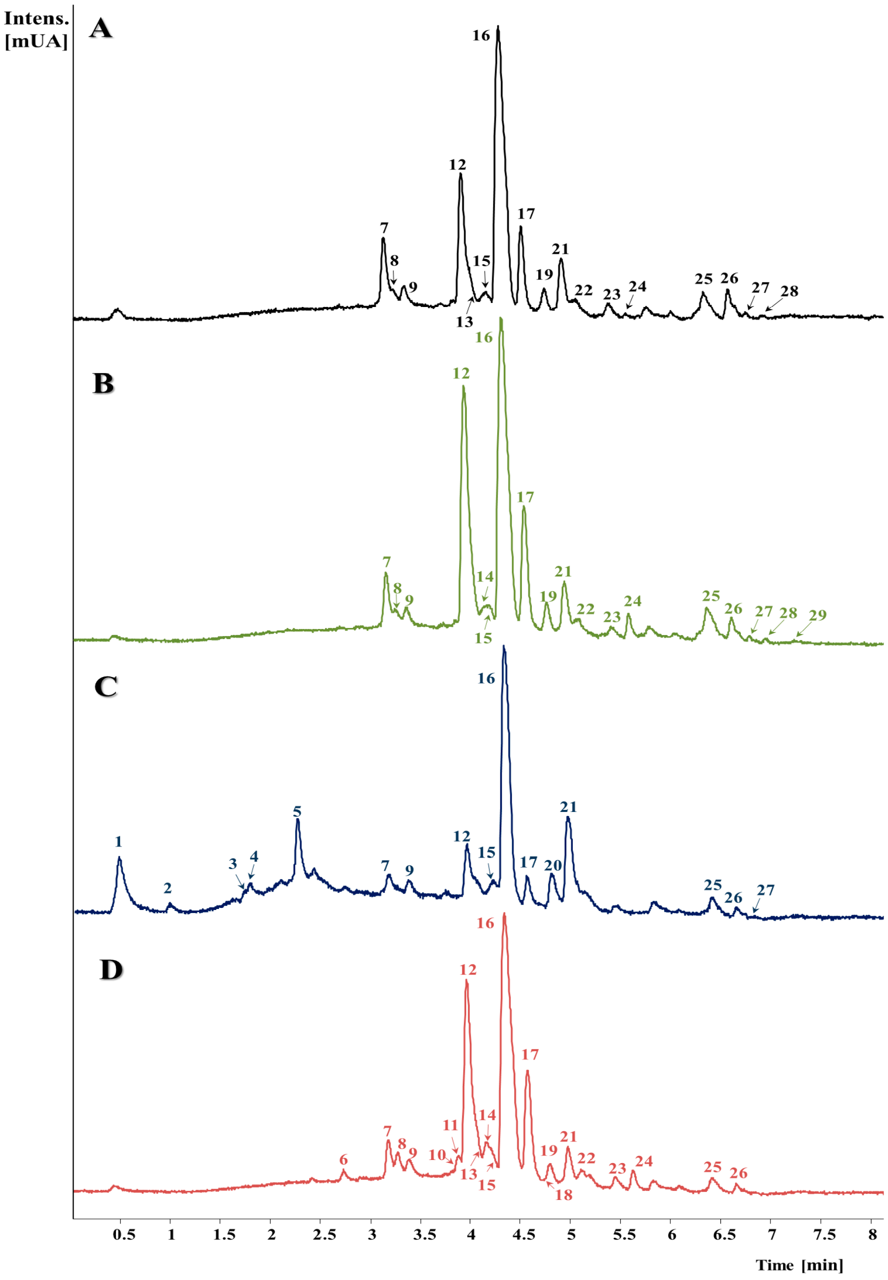

Figure S2. Chromatograms (at 280 nm) corresponding to SFE1 (**A**); SFE2 (**B**); PLE (**C**) and SAF1 (**D**) extracts. SAF1 was selected as example for PLE + SAF integrated process. For peak identification, see Table 3.
